# Part II—Corrosion Behavior and Mechanical Properties of Metallic Materials

**DOI:** 10.3390/ma19071323

**Published:** 2026-03-26

**Authors:** Xiaogang Li

**Affiliations:** Institute of Nuclear and New Energy Technology, Key Laboratory of Advanced Reactor Engineering and Safety of Ministry of Education, Tsinghua University, Beijing 100084, China; xiaogangli@mail.tsinghua.edu.cn

Globally, corrosion-related costs—including both protection measures and productivity losses—exceed USD 400 million annually [1]. Corrosion, driven by chemical and electrochemical reactions, often leads to the deterioration of mechanical properties and, ultimately, materials themselves [2]. Metallic components, which are integral to the oil, gas, marine, nuclear energy, fuel cell, medicine, and power generation industries, are particularly susceptible to severe corrosion [3,4]. This not only compromises their service life but also poses significant safety risks. In many cases, aggressive corrosive environments constrain the application of advanced metallic materials, even those with superior mechanical performance [5]. Therefore, understanding corrosion and its impact on the mechanical properties of metallic materials remains critically important for their development and reliable application. Both the mechanisms and resulting effects of corrosion on mechanical performance are strongly influenced by environmental conditions and a material’s physicochemical characteristics [6,7,8,9,10].

This Special Issue aims to serve as a dedicated forum for research on corrosion behavior and its correlation with the mechanical properties, chemical composition, and microstructure of metallic materials. The goal is to address current challenges that corrosion poses and to support the development of next-generation corrosion-resistant alloys. The topics of interest include, but are not limited to, the studies mentioned above. Other relevant studies, such as those related to hydrogen embrittlement, the characterization of the corroded microstructure, the corrosion mechanism of advanced materials, the method of surface treatment to improve corrosion resistance, the evolution mechanism of mechanical properties in a corrosive environment and the design of novel corrosion-resistant materials, will also be considered, which could enhance the knowledge of corrosion protection. As such, research articles and reviews in these areas of study are welcome.

Song et al. [7] contributed a paper entitled “Evaluation of Crack Formation in Heat Pipe-Welded Joints”, which revealed that the dual-insulated failure during heat supply resumption resulted from cracking along welded joints due to galvanic corrosion and thermal expansion-induced stress. This paper noted that the coarse-grained heat-affected zone was the most susceptible to corrosion and crack initiation, as it exhibited the lowest corrosion potential and anodic behavior. Furthermore, it was found that pre-existing microvoids in the heat-affected zone acted as critical stress concentrators, emphasizing the need for microstructural control in welded pipelines under corrosive and thermal stress.

Zhu et al. [Contribution 1] contributed a paper entitled “Molecular Dynamics Simulation on Orientation-Dependent Mechanical Behaviors of ZnO Single Crystals Under Nanoindentation”, in which the orientation-dependent mechanical behavior of ZnO single crystals was investigated under nanoindentation using a molecular dynamics simulation. The results reveal significant anisotropy in Young’s modulus, and plastic onset, with the a-plane ZnO exhibiting the earliest yielding and the highest modulus, and dislocation activities consistent with Schmid factor-predicted slip systems. These findings provide atomic-scale insights into the anisotropic deformation mechanisms of ZnO single crystals, which can inform the design and influence the reliability of ZnO-based devices.

Wang et al. [Contribution 2] contributed a paper entitled “Corrosion Performance in 0.5 mol/L HF Solution of Cr-Cu-Mo-Ni Porous Alloys with Varying Cr Contents”, in which porous Cr-Cu-Mo-Ni alloys (10–30 wt% Cr) were synthesized via activation reaction sintering, and their corrosion behavior in 0.5 M HF was evaluated. Among the porous alloys with different Cr contents, the 20 wt% Cr alloy exhibited optimal corrosion resistance, surpassing dense Ni/Cu alloys. This demonstrates that the corrosion process can be modulated via electrochemical reactions. Furthermore, it was found that the resulting corrosion products adhere to the inner surfaces of the pores, which contributes to improved corrosion resistance.

Żaba et al. [Contribution 3] contributed a paper entitled “Assessment of the Corrosion Rate of Maraging Steel M350 Produced by Additive Manufacturing Using the Laser Powder–Bed Fusion Method and Surface Finishing Techniques”, which reports how laser powder bed fusion parameters and surface finishing affect the corrosion behavior of M350 maraging steel. The key results are as follows: laser power has opposite effects on corrosion rates at room and elevated temperatures, and mechanical polishing alone cannot enhance corrosion resistance. The paper offers practical guidelines for optimizing additive manufacturing strategies to improve the reliability of M350 steel in chloride-containing environments.

Majchrowicz et al. [Contribution 4] contributed a paper entitled “Assessment of 10CrMo9-10 Power Engineering Steel Degradation State by Using Small Punch Test”, in which a small punch test framework to characterize mechanical property changes in service-aged 10CrMo9-10 steel was established, which correlated small punch test force parameters with tensile strengths via calibrated annealed reference samples. Validated on exploited steel, the small punch test-derived strength estimates aligned closely with conventional tensile test results, confirming its utility as a practical method for evaluating the mechanical performance of power engineering steel components.

Tang et al. [Contribution 5] contributed a paper entitled “Differential Corrosion Behavior of High-Aluminum 304 Stainless Steel in Molten Nitrate Salts: The Roles of Rolling and Heat Treatment”, which developed a low-cost Al-modified 304 stainless steel for concentrated solar power systems, achieving the enhanced corrosion resistance of molten salt due to the formation of a protective Al_2_O_3_–Cr_2_O_3_ composite oxide film. Corrosion tests at 600 °C reveal that cold rolling and annealing degraded the corrosion performance, while solution-treated hot-rolled high-aluminum 304 stainless steel showed superior resistance. The low-cost alloy developed in this study is highly promising as an alternative to 347H stainless steel for concentrated solar power applications.

Ma et al. [Contribution 6] contributed a paper entitled “Effect of Temperature on Corrosion of HSLA Steels with Different Cr Contents in a Water-Saturated Supercritical CO_2_ Environment”, which investigates the influence of Cr content and temperature on the corrosion behavior of steels in water-saturated supercritical CO_2_ environments, aiming to provide a fundamental basis for material selection and corrosion mitigation in S-CO_2_ pipeline systems. Increasing the Cr content refined the microstructure and resulted in the formation of a compact Cr_2_O_3_ layer that markedly improved the corrosion resistance, while a rising temperature shifted the corrosion mechanism from electrochemical to chemical control and reduced the overall corrosion rate. These results provide essential theoretical support for materials’ design, operational optimization, and corrosion protection in S-CO_2_ transport infrastructure.

Kawałek et al. [3] contributed a paper entitled “Formation of the Structure, Properties, and Corrosion Resistance of Zirconium Alloy Under Three-Roll Skew Rolling Conditions”. This paper investigated radial shear rolling, which produced an ultrafine-grained structure in Zr–1%Nb and significantly simplified fabrication compared with conventional processes. High-temperature steam corrosion tests confirmed that radial shear rolling a Zr–1%Nb alloy met the ASTM B351 standards and exhibited excellent corrosion resistance, validating radial shear rolling as an efficient and cost-effective thermomechanical route for nuclear-grade Zr alloys.

Geng et al. [Contribution 7] contributed a paper entitled “Multiscale Modeling Analysis of the Mechanical Behaviors and Failures of In Situ Particle Reinforced Titanium Matrix Composites Based on Microstructural Characteristics”, which proposed a multiscale finite element model to study the mechanical behavior and failure of TiB/Ti55531 titanium matrix composites. Molecular dynamics were used to calibrate the traction–separation response of the Ti/TiB interface for the cohesive zone model, which was then incorporated into a mesoscale heterogeneous representative volume element, enabling the quantitative analysis of stress transfer, damage initiation, and fracture mechanisms in the composites.

This Special Issue features innovative approaches and perspectives dedicated to elucidating the corrosion behavior and mechanical properties of metallic materials. We express our sincere gratitude to all the authors who have contributed manuscripts to this Special Issue and extend our appreciation to the readership at large. It is our aspiration that this Special Issue will serve as a scholarly platform for academic dialog and exchange among researchers in the field of metallic materials. Furthermore, in light of the sustained submission of high-quality papers on this theme, we have launched the third edition of this Special Issue, entitled “Corrosion Behavior and Mechanical Properties of Metallic Materials” (3rd Edition), with the submission portal available at https://www.mdpi.com/journal/materials/special_issues/4RA6FGO6J5 (accessed on 24 March 2026). The topics of interest encompass, but are not limited to, corrosion behavior, mechanical properties, chemical composition, microstructure, hydrogen embrittlement, the characterization of corroded microstructures, the corrosion mechanisms of advanced materials, surface modification strategies for enhanced corrosion resistance, the evolutionary mechanisms of mechanical properties in corrosive environments, and the design of novel corrosion-resistant metallic materials. Original articles and review papers within this research domain are welcomed.
